# On the impact of carbohydrate-binding modules (CBMs) in lytic polysaccharide monooxygenases (LPMOs)

**DOI:** 10.1042/EBC20220162

**Published:** 2023-04-18

**Authors:** Zarah Forsberg, Gaston Courtade

**Affiliations:** 1 Faculty of Chemistry, Biotechnology and Food Science, NMBU—Norwegian University of Life Sciences, 1432 Ås, Norway; 2 Department of Biotechnology and Food Science, NTNU—Norwegian University of Science and Technology, 7491 Trondheim, Norway

**Keywords:** carbohydrate binding modules, enzymology, lytic polysaccharide monooxygenase (LPMO), metalloenzymes, protein-carbohydrate interactions

## Abstract

Lytic polysaccharide monooxygenases (LPMOs) have revolutionized our understanding of how enzymes degrade insoluble polysaccharides. Compared with the substantial knowledge developed on the structure and mode of action of the catalytic LPMO domains, the (multi)modularity of LPMOs has received less attention. The presence of other domains, in particular carbohydrate-binding modules (CBMs), tethered to LPMOs has profound implications for the catalytic performance of the full-length enzymes. In the last few years, studies on LPMO modularity have led to advancements in elucidating how CBMs, other domains, and linker regions influence LPMO structure and function. This mini review summarizes recent literature, with particular focus on comparative truncation studies, to provide an overview of the diversity in LPMO modularity and the functional implications of this diversity.

## Introduction

Carbohydrate-binding modules (CBMs) are small non-catalytic protein domains that are part of modular enzymes, in particular carbohydrate-active enzymes (CAZymes). CBMs have been extensively studied because of their contribution to enhanced polysaccharide conversion by glycoside hydrolases (GHs) [1–3]. In biomass-degrading enzymes, the primary role of CBMs is to bind and direct the enzyme to its (poly)saccharide substrate and thereby increase the concentration of enzyme on the substrate surface, which is important as many of these enzymes work at a solid–liquid interface. As a result, many CAZymes are (multi)modular with catalytic function(s) provided by a single or multiple catalytic domains coupled to one or more CBM(s). The domains are connected by linker peptides of varying length and composition. Due to the complexity and vast number of polysaccharides in nature, there are numerous types of CBMs that are currently divided (based on sequence similarity) into 93 distinct families in the carbohydrate active enzyme database [1,4]—a number that has more than doubled in the past 20 years. CBMs are further grouped into three types based on structural and functional similarities, namely, ‘surface-binding’ CBMs (Type A), ‘*endo*-type’ CBMs that bind internally on glycan chains (Type B), and ‘*exo*-binding’ CBMs that bind to the termini of glycan chains (Type C) [1,5].

Lytic polysaccharide monooxygenases (LPMOs) are a group of redox active enzymes that have relatively recently been added to the CAZy database [6–9]. Such enzymes are found in families 9–11 and 13–17 of auxiliary activities (AAs; i.e., redox active CAZymes). LPMO activity is characterized by oxidization of the C1 and/or C4 carbon in (1–4)-linked polysaccharides, in a reaction that requires an electron donor and H_2_O_2_ as cosubstrate [10–12]. In addition, LPMOs exhibit low oxidase activity (i.e., reducing O_2_ to H_2_O_2_), a reaction that is more prominent in the absence of a (poly)saccharide substrate [13,14]. Unique to LPMOs is their large substrate binding surface which holds a single copper cofactor coordinated by two histidines in the catalytic center. Some of the first characterized LPMOs had a large, flat surface with features generally found in Type A CBMs, specifically planar aromatic residues that may stack with the (poly)saccharide substrate [15,16]. Due to these similarities bacterial LPMOs were first thought to be non-catalytic proteins belonging to the no longer existing family 33 of CBMs [17–19]. Today, it is well established that LPMOs are redox enzymes and important contributors to the efficiency of polysaccharide-degrading enzyme cocktails in industry and nature alike.

**Figure 1:**
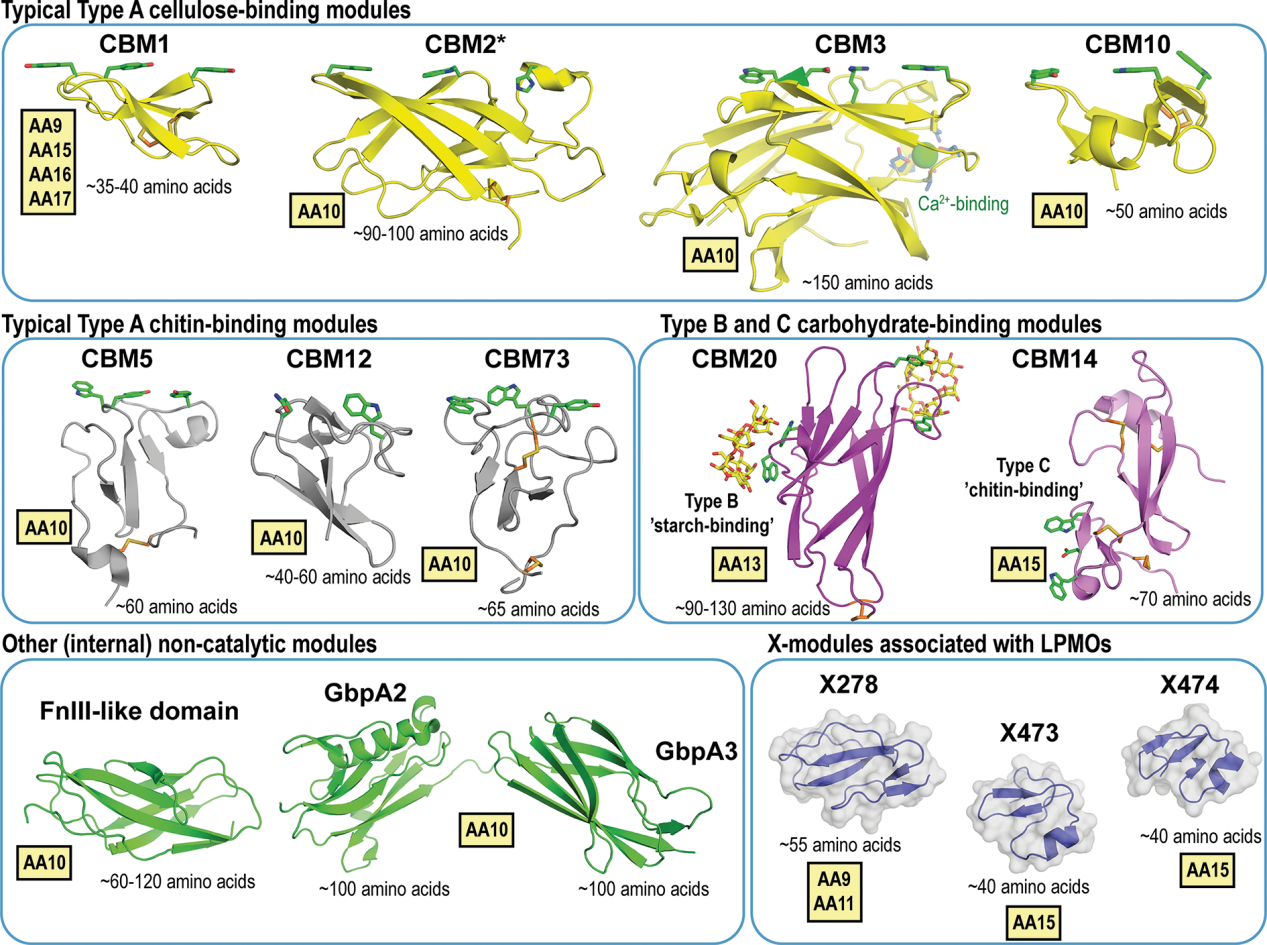
Common CBMs and other modules found in modular LPMO sequences CBM-truncation and/or substitution studies have been carried out for all Type A CBMs listed above, whereas Type B and C CBMs have only been observed, but not yet functionally studied, in LPMOs. The structures above illustrate representatives of CBMs from LPMOs and other CAZymes (in the absence of CBM structures from modular LPMOs) that belong to *Trichoderma reesei* Cel7A (CBM1, PDB 1CBH), *Streptomyces coelicolor* LPMO10C (CBM2, PDB 6F7E), *Acetivibrio cellulolyticus* cellulosomal scaffoldin ScaA (CBM3, PDB 3ZQW), *Cellvibrio japonicus* Xyl10A (CBM10, PDB 1ER8), *Cellvibrio japonicus* LPMO10A (CBM5, PDB 6Z40, PDB 6Z41), *Bacillus circulans* chitinase A1 (CBM12, PDB 1ED7), *Pseudocercospora fuligena* Avirulence protein 4 (Avr4) (CBM14, PDB 4Z4A), and *Aspergillus niger* glucoamylase granular starch-binding domain in complex with cyclodextrin (CBM20, PDB 1AC0). Internal modules belong to the FnIII-like domain from *Bacillus circulans* Chi18A (FnIII-like domain, PDB 1K85) and *Vibrio cholerae* LPMO10B/GbpA (GbpA2 and GbpA3, PDB 2XWX). The X-module structures are derived from AlphaFold2 models available at Uniprot (UniProtKB, Q2UA85 (X278), W4G2T6 (X473), and T0QAI8 (X474). * CBM2 can also bind chitin and are found in both chitinolytic and cellulolytic enzymes.

Like other CAZymes, LPMOs are found as single and (multi)modular enzymes. Importantly, in such multimodular enzymes the LPMO domain is always found at the N-terminus, as the N-terminal histidine is crucial for copper binding and thus activity. In fungal LPMOs of family AA9, which are active on cellulosic and hemicellulosic substrates, small (cellulose-binding) CBM1 domains are, according to Pfam (PF03443) [20] and characterization work of numerous AA9s, predominantly found associated with the catalytic domain. In contrast, a Pfam search on bacterial LPMOs from family AA10 (PF03067), active on chitinous and/or cellulosic substrates, reveal more diversity in terms of attached CBMs and commonly contain CBMs from families 2, 3, 5, 10, 12 and 73 (see Figure 1). The vast majority of CBMs connected to LPMOs belong to Type A CBMs, i.e., CBMs evolved to bind crystalline surfaces, although Type B CBMs have been observed in fungal starch-oxidizing AA13s [21,22] and Type C CBMs (‘chitin/chito-oligosaccharide-binding’ CBM14 and CBM18) have been found in family AA15s (CBM14; [23,24]) as well as in AA9s (CBM18), although the CBM18s have only been observed in connection to a domain of unknown function named X280 [25]. There are several so-called X modules, i.e., uncharacterized internal and terminal modules found in LPMOs and other CAZymes that may be yet undiscovered CBMs [24–26]. Furthermore, fibronectin type 3-like domains (FnIIIs) are found in bacterial LPMO sequences, but in contrast with CBMs, which are mostly found at the protein termini, FnIIIs are commonly located between other protein domains suggesting that they may function as spacer domains [27,28]. Similarly, the sequences of LPMOs from certain Gram-negative bacteria that resemble the *Vibrio cholerae* LPMO10B (GbpA, *N*-acetylglucosamine binding protein [29]), which is associated with bacterial virulence, have one or two internal modules of unknown function (named GbpA2 and GbpA3).

In this review, we compile studies on the structural and functional effects of CBMs and other domains associated with LPMO catalytic domains, with focus on the implications of CBMs on the autocatalytic inactivation of LPMOs. Furthermore, we touch upon the scarce work on linkers in modular LPMOs and discuss aspects to consider in future studies aimed at achieving stable and efficient LPMO reactions.

## Studies of CBM truncations

Since the discovery of LPMO activity in 2010 several CBM truncation studies have been carried out (see Table 1), confirming that CBMs attached to LPMOs have effectively the same function as CBMs in other biomass-degrading enzymes, namely promotion of substrate binding [30–40].

**Table 1:** Overview of domain truncation, CBM substitution and linker studies in LPMOs

Enzyme (regioselectivity, substrate specificity) wild-type (WT) modular organization	Key findings*	References
***Vc*LPMO10B (GbpA)** (C1-oxidizing, chitin) AA10-GbpA2-GbpA3-CBM73	Chitin-binding is mainly aided by the CBM and to a lesser extent by the LPMO domain.	Wong et al., 2012 [29]
	The LPMO domain is required for mucin binding and GbpA2 and GbpA3 in combination with the LPMO domain are important for intestinal colonization in a cholera mouse model.	
***Sc*LPMO10C** (C1-oxidizing, cellulose) AA10-CBM2	Loss of cellulose binding affinity for the CBM truncated enzyme.	Forsberg et al., 2014 [41]
	Reduced cellulose activity of CBM truncated enzyme.	Forsberg et al., 2014 [30]
	NMR spectroscopy study showing structural and dynamic features of a modular LPMO.	Courtade et al., 2018 [31]
	The substrate binding affinity resides with the CBM.	
	The CBM is beneficial for LPMO activity at lower substrate concentrations and promotes localized and repeated oxidation of the substrate.	
	Truncation of the CBM leads to elevated H_2_O_2_ production and decreased enzyme stability, both in absence and presence of cellulose.	Stepnov et al., 2022 [42]
***Nc*LPMO9C** (C4-oxidizing cellulose, cello-oligosaccharides, xyloglucan) AA9-CBM1	*K*_d_ measured for PASC and xyloglucan showed weaker binding for CBM truncated *Nc*LPMO9C.	Borisova et al., 2015 [43]
	No CBM truncation effect on activity on PASC but a 2-fold reduction in the rate of xyloglucan degradation.	
	Truncation of the CBM reduced the binding affinity and LPMO activity but did not affect regioselectivity.	Laurent et al. 2019 [38]
	The linker is important for thermal stability.	
***Cf*LPMO10** (C1/C4- oxidizing, cellulose and C1- oxidizing, chitin) AA10-CBM2	Study on deleting and replacing CBMs in two cellulose-oxidizing LPMOs.	Crouch et al., 2016 [32]
	Introduction of other types of cellulose binding CBMs (CBM2*^Tb^*, CBM3, CBM10) both potentiated and inhibited the LPMO activity. Such effects were both enzyme and substrate specific.	
	Changed ratios between non-oxidized and oxidized products when replacing the CBM2 by a CBM10 implies that CBMs can modulate the mode of action of LPMOs.	
***Tb*LPMO10** (C1-oxidizing, cellulose) AA10-CBM2	See above (*Cf*LPMO10).	Crouch et al., 2016 [32]
***Cj*LPMO10A** (C1-oxidizing, chitin) AA10-CBM5-CBM73	Removal of both CBMs reduced LPMO activity toward α-chitin compared with the full-length enzyme, but in synergistic reactions with an *endo*-chitinase equal levels of solubilized products were observed.	Forsberg et al., 2016 [34]
	Structural analysis of two similar chitin-binding CBMs with different affinity for crystalline chitin and soluble chitohexaose. CBM-containing variants performed better at low concentrations.	Madland et al., 2021 [35]
***Hj*LPMO9A** (C1/C4-oxidizing cellulose) AA9-CBM1	Removal of the CBM, post-translationally by papain hydrolysis, led to a truncated variant with 21 remaining residues of the predicted linker which exhibited reduced binding and activity towards cellulose compared with the full-length enzyme. The X-ray structure revealed that the glycosylated linker forms an integral part covering a hydrophobic patch on the catalytic LPMO domain.	Hansson et al., 2017 [44]
	Removing the CBM resulted in reduced binding but did not alter the oxidative regioselectivity. However, the effects of point mutations (Y24A, Y211A and Y24A_Y211A) in the catalytic domain on oxidative regioselectivity became more apparent in the absence of the CBM.	Danneels et al., 2019 [39]
***Tf*LPMO10B** (C1-oxidizing, cellulose) AA10-FnIII-CBM2	Binding is mediated mainly by the CBM and to some extent by the LPMO domain. Removal of the FnIII-like domain (called X1) had no effect on binding nor on activity.	Kruer-Zerhusen et al., 2017 [40]
***Ma*LPMO10B** (C1/C4- oxidizing, cellulose and C1- oxidizing, chitin) AA10-CBM2	Deletion of the CBM affected the operational stability of the LPMO but did not affect the ratio of regioselective C1:C4 oxidation.	Forsberg et al., 2018 [33]
***Bc*LPMO10A** (C1- oxidizing, chitin) AA10-FnIII-FnIII-CBM5	Enzyme functionality was strongly dependent on the CBM that is responsible for substrate binding and protects the enzyme from autocatalytic inactivation. Truncation of one or two of the FnIIIs (both in combination with the CBM) resulted in essentially the same effect as when only the CBM was removed.	Mutahir et al., 2018 [36]
***Bt*LPMO10A** (C1-oxidizing, chitin) AA10-FnIII-FnIII-CBM5	The CBM is essential for binding to α- and β-chitin. The FnIII-like domains do not have a role in chitin-binding.	Manjeet et al., 2019 [45]
***Pa*LPMO9H** (C1/C4-oxidizing cellulose, cello-oligosaccharides, xyloglucan) AA9-CBM1	Truncation of the CBM weakened substrate binding and affected the catalytic performance on nanofibrils, amorphous and crystalline cellulosic substrates, but there was no effect on the activity on cellohexaose.	Chalak et al., 2019 [37]
	Increasing the substrate concentration reduces the need for a CBM.	
	The truncated variant showed a modified regioselectivity with increased C1-oxidation.	
	Optical and atomic force microscopy of the insoluble fraction revealed that both variants can promote disruption of the cellulose network and that the CBM is not essential.	
***Jd*LPMO10A** (C1-oxidizing, chitin) AA10-CBM5-GH18	Synergy study that showed intramolecular synergy between the LPMO domain and the chitinase (GH18) domain.	Mekasha et al., 2020 [46]
	Comparison of the chitinolytic efficiency of the full-length enzyme and combinations of truncated variants showed that the full-length enzyme is more efficient compared with any combination of its separately produced domains.	
***Mt*LPMO9B** (C1-oxidizing, cellulose) AA9-CBM1	The CBM promotes cellulose degradation in the full-length enzyme but does not affect oxidative regioselectivity.	Sun et al., 2021 [47]
***Bc*LPMO9C** (Unknown regioselectivity, cellulose) AA9-CBM1	Studies on linker truncation showed that shortening the linker or removing the CBM reduces substrate binding.	Srivastava et al., 2022 [48]

*Note that the more quantitative statements in this table need to be read with caution because the quality of the underlying kinetic analyses varies. For example, especially in early studies, the impact of autocatalytic LPMO inactivation, leading to nonlinear progress curves, was not always considered [49].

An updated version of this table is also available online at https://github.com/gcourtade/papers/tree/master/2022/LPMO-modularity-review.

Early work demonstrated that removal of the CBM2 from *Sc*LPMO10C resulted in loss of cellulose binding [41]. In a contemporary study, the same truncation was also found to diminish the release of oxidized sugars over time [30], a result that later was found to be a consequence of reduced lifetime of the catalytic domain due to low or insufficient substrate binding [10,33,50,42]. Similar truncation studies have been performed on bacterial LPMOs with different oxidative regioselectivities [32,33], substrate specificity [34,36], and on fungal LPMO9s [37,39,47].

In 2016, Crouch et al. presented a comprehensive study in which the CBM2s in two bacterial cellulose-oxidizing LPMOs (*Cf*LPMO10 and *Tb*LPMO10; see Table 1) were deleted or replaced with other types of bacterial cellulose-binding CBMs (i.e., CBM2, CBM3 and CBM10; see Figure 1) [32]. They found that introducing other types of CBMs both potentiated and inhibited the LPMO activity and that such effects were both enzyme and substrate specific. For example, when the CBM2 was replaced by the CBM10, the activity for both LPMOs was substantially higher when Avicel was used as a substrate, whereas appending the CBM with the highest affinity for all three tested celluloses (i.e., CBM2 from *Tb*LPMO10) was optimal for product formation in reactions with phosphoric acid swollen cellulose (PASC) and bacterial microcrystalline cellulose (BMCC). Adding a CBM with similar affinity as *Tb*CBM2, namely CBM3 from the cellulosomal protein CipA which is responsible for anchoring the cellulosome of *Clostridium thermocellum* to cellulose, resulted in reduced or completely abolished LPMO activity, suggesting that the CBM3 directed the LPMO to regions that are inaccessible for the catalytic domain. Furthermore, it was shown that fusing the LPMOs with a CBM10 led to a change in the ratio between oxidized and non-oxidized products. While this early study did not fully take into account the complexities of assessing LPMO activity, such as enzyme inactivation [49], the work of Crouch et al. showed that CBMs can direct and alter LPMO activity [32].

Oxidative regioselectivity of cellulose-active LPMOs (C1 or C4 oxidizing), is determined by the binding mode of the LPMO on the substrate [43,51]. As the CBM affects how the LPMO domain interacts with the substrate, a few studies have addressed the effect of CBMs on LPMO regioselectivity. Removal of the CBM1 from *Pa*LPMO9H, an enzyme with mixed C1/C4 activity caused a shift in the ratio between C1- and C4-oxidized products, with the CBM-free enzyme generating an increased fraction of C1-oxidized cello-oligosaccharides [37]. In contrast, data presented by Danneels et al. indicated that the regioselectivity of C1/C4-oxidizing *Hj*LPMO9A was unaffected upon removing the CBM1, although the effects of point mutations in the catalytic domain became more apparent in the absence of the CBM [39]. Likewise, no difference in regioselectivity was observed upon truncation of the CBM in C1/C4-oxidizing *Mt*LPMO9B and *Ma*LPMO10B [33,47]. These seemingly contradictory observations indicate that more data is needed to adequately elucidate the effect of CBMs on LPMO regioselectivity.

While several studies have reported that recombinantly expressed isolated catalytic domains of otherwise CBM-containing LPMOs bind weakly to the substrate [34,36,38,41,43], some naturally occurring single domain LPMOs bind strongly to their substrate [18,52–54]. These latter proteins are all chitin-active, binding studies for natural single domain cellulose-active LPMOs are still scarce. Notably, several well-studied and higher performing cellulose-active AA9 LPMOs, which are likely industrially used, are in fact single domain proteins [55,56]. These observations suggest that weak binding by the catalytic domain is compensated by attachment to a CBM; however both LPMO–substrate and CBM–substrate interactions are relevant for understanding binding affinities. Crystallographic studies with soluble, non-crystalline oligosaccharides have provided mechanistic insights on critical LPMO–cello-oligosaccharide interactions [51,57,58]. However, CBMs are less important for activity on soluble substrates and more crucial for targeting crystalline cellulose [38,43,59]. The latter is supported by findings reported by Chalak et al., who showed enhanced performance of CBM1-containing *Pa*LPMO9H on polymeric cellulose substrates compared with the isolated catalytic domain, while the advantage of the CBM1 was less important when cellohexaose was used as the substrate [37]. Importantly, substrate binding by LPMOs is affected by the redox state of the enzyme [60,61]. For example, ascorbic acid-reduced *Nc*LPMO9C exhibited a lower dissociation constant for PASC (*K*_d_ = 4.4 ± 1.0 μM) compared with the oxidized LPMO (*K*_d_ = 9.5 ± 2.2 μM) [60] and this effect was noticeable despite the presence of a CBM that probably contributes heavily to substrate affinity.

## Studies of multimodular LPMOs

A few studies have focused on multi-modular LPMOs (i.e., enzymes containing more than a single CBM in addition to the LPMO catalytic domain) with the aim of understanding the roles of the CBMs and other domains, as well as the interplay between these. One of these enzymes is the tri-modular *Cj*LPMO10A, possessing an N-terminal AA10 followed by an internal CBM5 and a C-terminal CBM73 domain, both being chitin-specific CBMs [34,35]. In 2021, Madland et al. demonstrated that the two seemingly similar CBMs differ as the CBM73 has higher affinity for crystalline chitin than the CBM5. Further highlighting the difference, NMR titration experiments showed that the CBM5, as opposed to the CBM73, can bind soluble chitohexaose. Truncation of both CBMs led to rapid inactivation of the enzyme under turnover conditions, but removal of only the CBM73 showed no discernible differences in catalytic behavior compared with the wild-type enzyme [35]. In an earlier study of the same enzyme, synergism with an *endo*-chitinase was compared for full-length *Cj*LPMO10A and its lower performing isolated catalytic domain, which remarkably revealed that there were no differences in the boosting effect of the LPMO on chitinase activity [34]. Such results are in line with a study by Courtade et al. showing that removal of the CBM leads to different product spectra (see below). Briefly, lack of the CBMs leads to a more random oxidation pattern, which entails that a higher fraction of oxidized sites will remain in the insoluble fraction (hence lower activity in terms of the release of soluble products). Such oxidized sites will be solubilized in reactions also containing a GH, thus diminishing the difference in solubilization yields between the full-length and the CBM-truncated enzyme.

Synergy with GHs is what also makes LPMOs of exceptional interest for biomass deconstruction [19,55]. Interestingly, some LPMO domains are part of bicatalytic enzymes that contain an additional catalytic CAZyme domain [62,63]. To date only one of these enzymes has been characterized, namely *Jd*1381 possessing an N-terminal AA10 module followed by a CBM5 and a GH18 chitinase [46]. The full-length enzyme and four truncated versions (see Table 1) were subjected to a series of synergy experiments followed by quantification of non-oxidized (primarily produced by the GH18) and LPMO-oxidized products. The results showed that the full-length enzyme was more efficient in degrading the substrate compared with any combination of its separately produced domains, indicating that the full catalytic potential was only harnessed when the two catalytic units were covalently linked [46]. These results align with work showing that integration of two bacterial LPMOs in a designer cellulosome, alongside an *endo*- and an *exo*-cellulase, increased cellulose conversion compared with a system containing the free enzymes [64]. Furthermore, this study revealed that the beneficial effect of assembling multiple activities in a cellulosome was stronger for an LPMO-containing enzyme system compared with an enzyme system lacking LPMOs [64].

## Additional non-CBM modules and intrinsically disordered regions

FnIII-like domains are evolutionary conserved and generally involved in protein–protein interactions, and as a spacer or ‘structured linker’ module shaping spatial arrangements of protein domains [65]. However, in LPMOs and other CAZymes, the role of FnIII-like domains remains unclear [27]. Recently, three studies have attempted to shed light on the functional role of FnIII-like domains in LPMOs. Mutahir et al. demonstrated that, whereas removal of the CBM reduced binding and activity of the tetra-modular *Bc*LPMO10A (Table 1), removal of the CBM together with one or two FnIII’s had no further effect on the function of the enzyme [36]. Similar findings have been reported by Manjeet et al for chitin-active *Bt*LPMO10A [45] and by Kruer-Zerhusen et al. for cellulose-active *Tf*LPMO10B [40], suggesting that FnIII-like domains do not interact with the carbohydrate substrate or influence catalytic activity but instead function as structural spacers between the CBM and the catalytic domain.

Similarly, no direct chitin-binding was observed [65] by Wong et al. for the two internal domains (GbpA2 and GbpA3, Figure 1) in the *Vibrio cholerae* LPMO known as GbpA. However, the two domains, being distant structural homologs of bacterial pili binding proteins, were shown to be important for binding to the *V. cholerae* surface to potentiate a stable interface between the bacterium and the host that facilitates colonization. Knockout studies demonstrated that GbpA2 and GbpA3 in combination with the LPMO domain (but not the CBM) are required for colonization of intestinal epithelium in a cholera mouse model, whereas the CBM is important for chitin-binding [29]. Considering the potential protein interaction functions of FnIII’s and GbpA2/A3 as well as the recent findings of common C-terminal extensions (see below) in certain LPMO subfamilies [26], it is conceivable that LPMO-appended domains do not exclusively bind to carbohydrates.

In a recent bioinformatics study by Tamburrini et al., it was discovered that a considerable fraction (∼60% of 27,060 sequences) of LPMOs from all families, except for AA13s, possesses an intrinsically disordered region (IDR) at the C-terminus. Such C-terminal extensions are generally longer than inter-domain linkers and have not been encountered in other CAZymes or oxidoreductases [26]. Due to the amino acid compositions (enriched in charged, polar and Gly/Pro, but lacking aromatic and hydrophobic amino acids), IDRs fail to adopt single well-folded structures but are rather described by an ensemble of conformations. IDRs appear to be functionally relevant as more than 70% of the LPMO C-terminal extensions contain at least one putative binding site that may bind to a partner and/or ligand and function in molecular recognition. Another suggested possible role of such IDRs is to mediate attachment to the cell wall [26], possibly similar to the attachment of GPI anchors that have been predicted for members of AA9, AA14, AA15, AA16 LPMOs and X325 LPMO-like proteins [66], or cell wall anchoring through a hydrophobic C-terminal tail with the LPXTG anchoring motif found in some AA10 LPMOs [67]. IDRs are generally removed during cloning of LPMOs and the specific functions of IDRs in LPMOs remain to be elucidated.

## Linkers in LPMOs

Even though linkers are important in regulating interactions between folded domains [68,69] and affect the overall shape of (multi)domain proteins, there is only limited knowledge regarding the structural and functional role of linkers in LPMOs. The few studies on LPMO linkers have mainly focused on characterizing the structural features that the linker confers to the full-length protein, whereas the potential role of O-glycosylation of serine and/or threonine residues in the linker, which may occur both in fungal and certain bacterial LPMOs sequences [70,71], has also been addressed to some extent.

Courtade et al studied the structural role of the linker in *Sc*LPMO10C (Table 1), which functions as a flexible tether between the LPMO and the CBM while allowing independent motions of the two domains [31]. NMR data for the linker region indicate that it exists in an extended conformation [72]. Structural information was also obtained for the linker region of fungal *Tt*LPMO9H using small-angle X-ray scattering (SAXS). Here, Higasi et al. determined that the *O*-glycosylated linker is flexible and slightly extended [73]. Moreover, *O*-glycosylation in the linker region of fungal *Hj*LPMO9A has been shown (PDB: 5O2X) to mediate tight binding of the linker to the catalytic domain [44].

Srivastava et al recently investigated the role of the linker region of fungal *Bc*LPMO9C (Table 1) focusing on its effect on catalytic performance and thermostability. The authors generated three linker truncation variants, where shortening of the linker seemingly resulted in lower cellulose-binding affinity [48].

The variability in linker length and composition across LPMOs, along with their effect on structure and function requires further investigation. Drawing on knowledge obtained for GHs [74], it would appear that evolutionary pressure governs conservation patterns in linkers. This evolutionary pressure is likely related, but not limited to, optimal enzyme functionality. Linker features such as *O*-glycosylation sites could also affect thermostability and resistance to proteolysis [75,76].

These insights have implications for protein engineering efforts aiming to fuse CBMs or other domains to LPMOs. The functional and structural roles of the linker (determined by its length and composition) should be considered when optimizing compatibility between the LPMO and the appended domain(s).

## CBM effect on product profiles and autocatalytic inactivation

**Figure 2:**
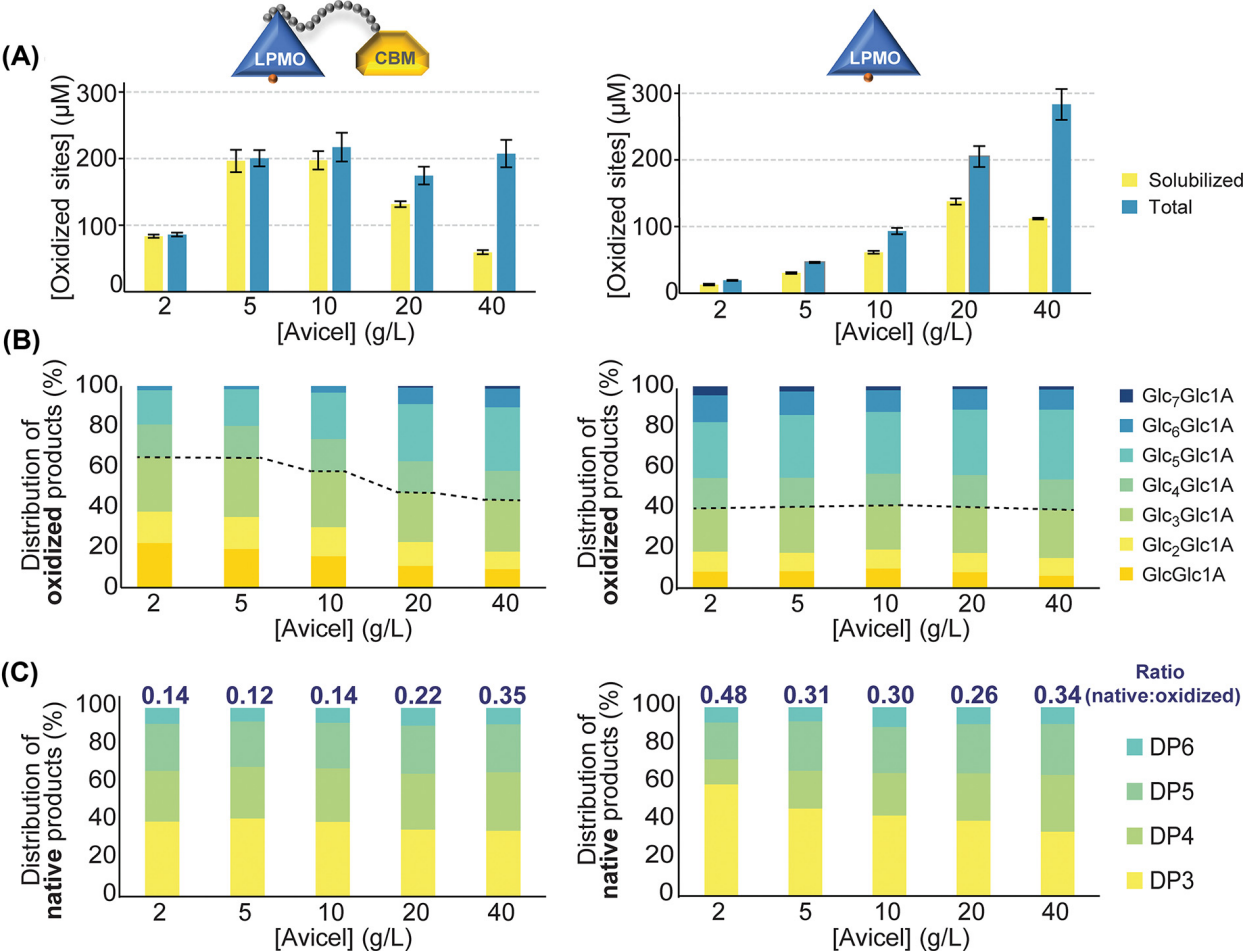
Generation of oxidized products by full-length *Sc*LPMO10C (left) and its isolated catalytic domain (right) Panel (**A**) shows solubilized and total oxidized (i.e., soluble and insoluble fraction) products at different substrate concentrations. Panels (**B,C**) show the relative distribution of oxidized and native (i.e., non-oxidized) products, respectively, with a degree of polymerization (DP) of 2–8 at varying substrate concentrations. The dotted lines in panel (**B**) indicate how the product distribution for the full-length enzyme shifts from a ‘localized’ to a more ‘randomized’ oxidation pattern in a substrate concentration dependent manner, a trend that is not observed for the isolated catalytic domain. The numbers above the bars in in panel (C) indicate the ratio between native products and oxidized products released by the two enzyme variants at different substrate concentrations. The figure was adapted from Courtade et al., 2018 [31].

Recent work by Courtade et al. and Stepnov et al. has shown that the roles of CBMs in modular LPMOs are considerably more intricate than just promoting substrate binding [31,42]. In Courtade et al., we showed that full-length *Sc*LPMO10C, as opposed to its isolated catalytic domain, carried out localized surface oxidation. Comparative functional studies at low substrate concentrations revealed significant differences that were explained by CBM-mediated binding to internal positions on the substrate surface, which promote multiple cleavages in the same region. This is reflected in the full-length enzyme solubilizing more products, relative to the fraction of insoluble products (Figure 2A), and the soluble products were generally shorter compared with products released by the isolated catalytic domain (Figure 2B). Another indicator for localized substrate oxidation was the ratio between oxidized and non-oxidized products, the latter of which can only emerge from LPMO cleavage close to chain ends. The fraction of non-oxidized products was considerably lower for the full-length enzyme (Figure 2C) signifying that the CBM binds and promotes activity at internal positions on the crystalline surface.

**Figure 3:**
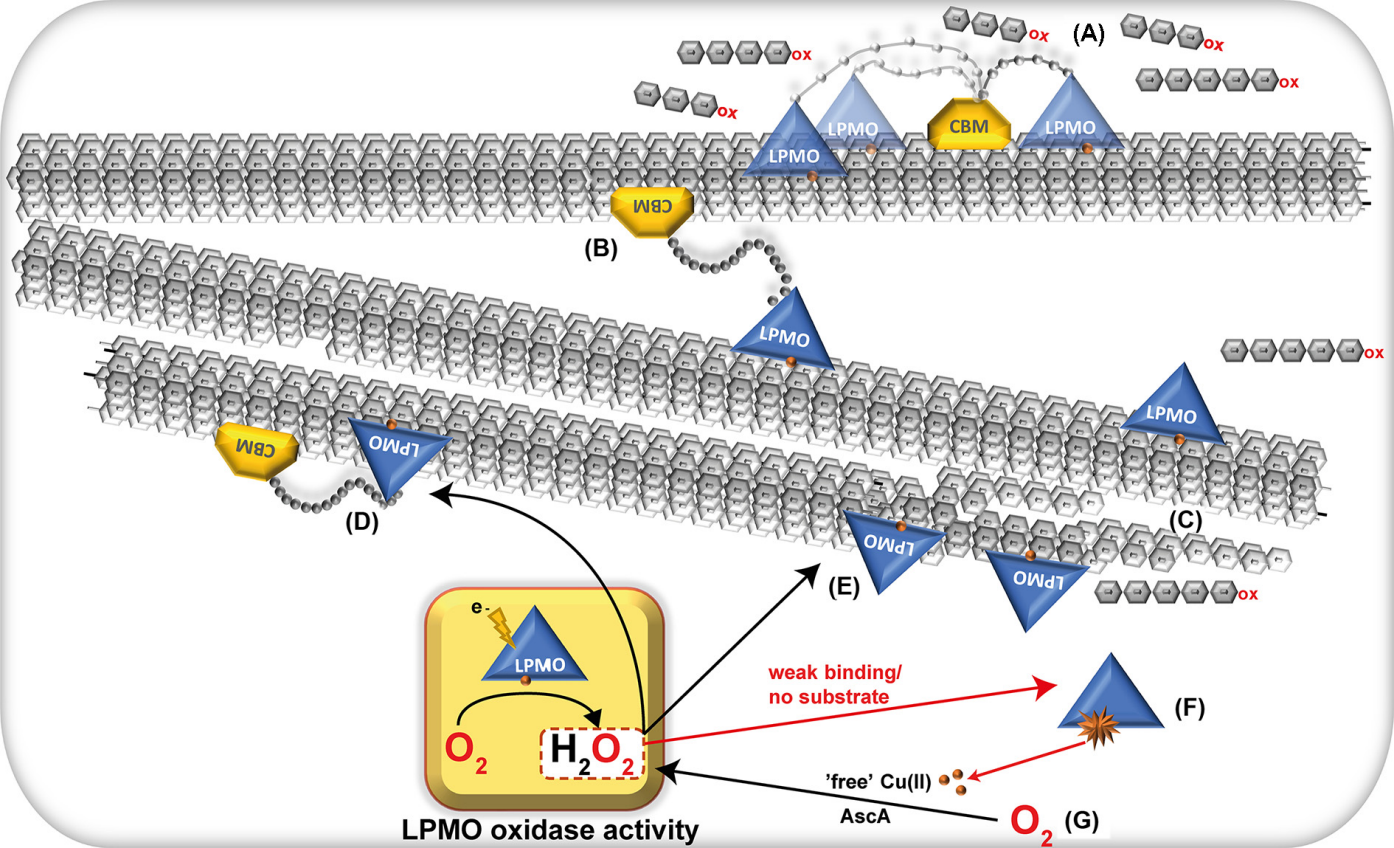
The impact of CBMs on LPMO activity Panel (**A**) shows an example of a modular LPMO with a catalytic domain (blue) connected to a CBM (yellow) via a flexible linker (grey dots) and illustrates how the CBM can dock the enzyme to the substrate followed by several oxidation events in the surrounding area carried out by the catalytic domain, which is restricted by the length and flexibility of the linker. The figure shows three variants shifting from compact to extended conformations [31]. This modular arrangement increases the chance to oxidize the same cellulose chain twice and thereby increases the release of short, soluble oligomeric products. (**B**) At higher substrate concentrations, the oxidation pattern becomes more random as the CBM may bind to one cellulose chain while the catalytic domain may oxidize another chain. Thus, the product pattern becomes more similar to that of (truncated) CBM-free LPMOs, illustrated in (**C**), which more rarely generate soluble products by cutting the same chain twice or by cutting close to a chain end. As a result of the weaker binding, such LPMOs will spend more time in solution as opposed to CBM-containing LPMOs, which leads to higher oxidase activity (as illustrated in the yellow box) and generation of H_2_O_2_, which may fuel the LPMO reaction (**D,E**) or be consumed in an off-pathway reaction (indicated by red arrows) leading to oxidative damage followed by inactvation (**F**). The oxidative damage is documented to mainly affect the catalytic histidines [10,50], causing leakage of copper from the active site followed by accelerated H_2_O_2_ production (**G**) [80] that consequently speeds up the processes in (**D–F**). The latter has recently been described by Stepnov et al. as a self-reinforced inactivation event [42].

In the study by Courtade et al., we also showed that while the CBM is crucial for prolonged activity at low substrate concentrations, removal of the CBM was demonstrated to be beneficial for the enzyme performance at higher substrate concentrations (Figure 2A), similar to what was first observed by Várnai et al. for CBMs in cellobiohydrolases [77]. Mutually, these findings suggest that there is a trade-off between beneficial CBM-mediated substrate affinity and possible negative effects of the CBM related to low off-rates and/or non-productive binding [31,77,78]. Chalak et al. obtained similar results when comparing CBM1-truncated *Pa*LPMO9H to its full-length variant at elevated substrate concentrations [37]. Consequently, it is not unexpected that some higher performing single modular LPMO9s, used in enzymatic cocktails, perform well in industrial settings where the substrate concentrations are usually high [55,56]. Increasing the substrate concentration in experiments with full-length *Sc*LPMO10C also revealed that the tendency of performing localized oxidation disappeared as the oxidation pattern became equally ‘random’ as for the catalytic domain (Figure 2A), and the size distributions of solubilized oligosaccharides appeared essentially the same for the two variants (Figure 2B). While the similar overall activities can be explained by the compensatory effect of a high substrate concentration on weaker substrate affinity (as for the catalytic domain), this effect does not explain the changes in the product profiles. It was suggested that at higher substrate concentrations the freely moving LPMO domain of a CBM-bound enzyme can act on a cellulose chain in another fibril to which it is not directly bound (Figure 3B). This would lead to more randomized and less localized cleaving, similar to what is expected for the catalytic domain only. Similarly, although not being a CBM truncation study, Koskela et al. showed that CBM-lacking *Nc*LPMO9F exhibited a pattern of less localized oxidation compared with *Nc*LPMO9E, which is tethered to a CBM1. Moreover, the CBM-lacking enzyme was more efficient in oxidizing the cellulose fibre surface, whereas *Nc*LPMO9E solubilized the fibre more efficiently. It was suggested that the absence of a CBM led to enhanced movement of the enzyme, allowing for oxidation in a more disperse manner over the entire cellulose surface [79].

**Figure 4:**
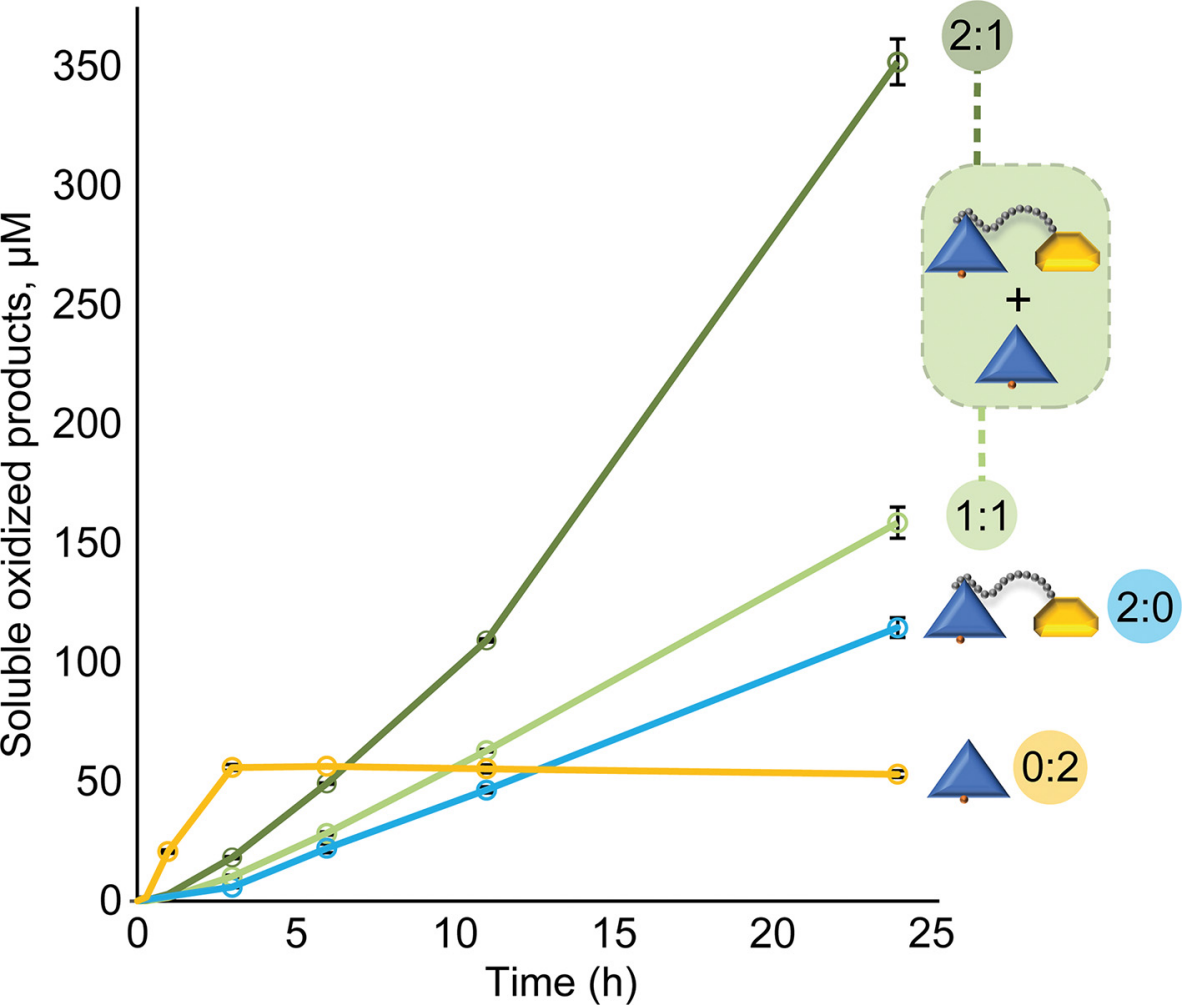
Cellulose oxidation by truncated and full-length *Sc*LPMO10C in separate or combined reactions The enzyme ratio (full-length: truncated) is indicated in the colored circles for all reactions. The figure was adapted from Stepnov et al., 2022 [42].

It is well known that LPMOs tend to lose activity under turnover conditions and that their operational stability may be low. It was shown in 2017 that this is due to autocatalytic inactivation [10]. LPMOs that are reduced and meet O_2_ or H_2_O_2_ while not being bound to a substrate are particularly prone to such autocatalytic inactivation (Figure 3), due to the reactivity of the reduced Cu(I) ion in the LPMO active site [10,12,61,81]. Consideration of this risk of inactivation and the emerging notion that LPMO activity in most typical reaction set-ups is limited by the level of *in situ* generated H_2_O_2_ [42,80,82] sheds additional light on the role of CBMs in LPMO catalysis. On the one hand, a CBM will contribute to the LPMO catalytic domain on average being closer to its substrate, thus increasing the chance that encountered H_2_O_2_ is used productively [31]. On the other hand, the oxidase activity of the LPMO (Figure 3), which, at least for some LPMOs is an important contributor to *in situ* generation of H_2_O_2_, may be hampered by substrate binding [83]. These effects and their consequences were effectively illustrated in a recent study by Stepnov et al., which showed that truncation of the CBM in *Sc*LPMO10C results in increased H_2_O_2_ production, higher LPMO activity and higher LPMO inactivation compared with the full-length enzyme (Figure 4) [42]. The wild-type enzyme, which is substrate-bound to a larger extent, is less active but does not suffer from inactivation (i.e., it shows steady product release over time limited by low H_2_O_2_ production, Figure 4). Interestingly, combining the two enzyme forms led to strong synergistic effects likely because H_2_O_2_ generated by the truncated variant is productively used by the substrate-bound full-length enzyme (Figure 4). Thus, cellulose degradation became faster, while enzyme inactivation caused by off-pathway reactions with excess H_2_O_2_ was reduced.

Another support for efficient and productive (CBM-mediated) H_2_O_2_ consumption was illustrated when the CBM1 was removed from pyranose dehydrogenase (PDH), an LPMO redox partner/H_2_O_2_ producer, which resulted in lower LPMO catalytic rates compared with reactions with full-length PDH. Therefore, it is conceivable that, in addition to vicinity to the substrate, LPMO activity may also be enhanced by CBMs by increasing the proximity between the substrate and the source of H_2_O_2_ [84].

## Summary

- LPMOs appended to CBMs generally perform better than single-domain LPMOs (i.e., truncated or naturally occurring) in dilute systems. This enhanced performance relates to increased operational stability (i.e., less autocatalytic inactivation), which is achieved through the close proximity of substrate and LPMO, increasing the chances for available H_2_O_2_ being used in productive reactions.
- The effect of CBMs on LPMO efficiency is dependent on the substrate concentration. At low concentrations the CBM is important to avoid off-pathway processes, but at high concentrations there are negative CBM-related effects such as low off-rates and/or non-productive binding.
- The effects of CBMs on *in situ* H_2_O_2_ production and the extent of off-pathway reactions need to be considered when developing cellulolytic enzyme cocktails, possibly containing multiple LPMOs.
- Along with CBMs, additional modules (e.g., FnIII-like domains), linkers and C-terminal extensions also have important roles in (multi)modular LPMOs that should be considered when engineering and fine-tuning LPMO performance.

## Abbreviations

BMCC: bacterial microcrystalline cellulose
CBM: carbohydrate-binding module
FnIII: fibronectin type 3-like domain
IDR: intrinsically disordered region
GH: glycoside hydrolase
LPMO: lytic polysaccharide monooxygenase
PASC: phosphoric acid swollen cellulose
SAXS: small-angle X-ray scattering
